# Osteitis Condensans Ilii: A Case Report

**DOI:** 10.7759/cureus.47504

**Published:** 2023-10-23

**Authors:** Samiksha D Lokhande, Nareshkumar Dhaniwala

**Affiliations:** 1 Orthopedic Surgery, Jawaharlal Nehru Medical College, Datta Meghe Institute of Higher Education and Research, Wardha, IND

**Keywords:** osteitis condensans ilii, sacroiliac joint, self-limiting, sclerosis, noninflammatory

## Abstract

Hyperostosis triangularis ilii, also called osteitis condensans ilii (OCI), is a rare condition, mostly occurring in females, and the etiology is unknown. This disease is a sclerotic disorder associated with iliac fibrosis, a noninflammatory and self-limiting disorder. This condition primarily affects the iliac part of the sacroiliac joints and sometimes the adjoining bones, such as the sacrum, lumbar vertebrae, and iliac bones. It is an incidental finding in many individuals but rarely associated with lower back pain due to sacroiliac joint involvement. It is mainly treated with physical therapy and medications. OCI should be considered a cause of chronic back pain in females not having ankylosing spondylitis or inflammatory arthritis. Herein, a case of osteitis condensans ilii in a 47-year-old female, presenting with the chief complaints of intermittent lower back pain in the midline and on both sacroiliac regions, without any evidence of ankylosing spondylosis and the X-ray of the pelvis and magnetic resonance imaging (MRI) showing features of OCI, is being reported. The case was managed with medications and exercise, and the patient is under regular follow-up.

## Introduction

Osteitis condensans ilii (OCI), also called osteopathia condensans ilii or hyperostosis triangularis ilii in German, is characterized by benign, triangular-shaped sclerosis of the ilium next to the sacroiliac joint. Although the underlying cause is unknown, it is thought that mechanical tension and imbalance across the sacroiliac joints are to blame, which leads to a long-lasting stress reaction. This theory is supported because it most frequently affects postpartum females. Sicard, Gally, and Haguenau initially characterized it in 1926 [1]. It is a self-limiting condition found incidentally on X-rays in asymptomatic patients or those presenting with lower back pain. The findings are localized to the sacroiliac joints, and the condition must be differentiated from spondyloarthropathy, osteoarthritis, or low-grade infective arthritis. OCI is a distinct entity because it does not involve the joint space, is not progressive, and commonly presents without any abnormal laboratory findings, even though some presenting characteristics may be similar to arthritis. Thus, it is confused with osteoarthritis of the sacroiliac joints [2]. OCI is predominantly seen in pregnant or recently delivered females of reproductive age, though this entity has been described in males and nulliparous females also [3]. It manifests in chronic back and hip discomfort [4]. OCI is primarily asymptomatic, although, in a few cases, it presents as early-onset low back pain that resembles axial spondyloarthropathy (SpA) [3]. Herein, a case of atypical bilateral OCI is reported due to its presentation at the age of 36 years, pain starting one year after delivery and persisting in a recurrent manner.

## Case presentation

A 47-year-old female presented in an orthopedic outpatient department (OPD) with chief complaints of pain in the lower back for 10 years. The pain started spontaneously in the center of the lower back and sacroiliac area without any radiation. It is mild in intensity and gets relieved spontaneously at rest but reoccurs at variable intervals. It increases on exertion and bending forward. It is present occasionally, but the pain in the middle of the lower lumbar spine is persistent and interferes with the day-to-day activities of the patient. The patient also experiences pain in the upper lateral aspect of both thighs while sitting cross-legged. The severity of the pain increases during menstruation. There is no history of morning stiffness, difficulty in back and neck movements, any other joint involvement, loss of weight or appetite, or trauma to the lower back. The patient is multiparous, having two children, and has a history of one abortion and two miscarriages. The last pregnancy was 11 years ago, and she started experiencing pain after one year. Her menstrual cycle is regular at an interval of 25 days and lasts 3-5 days with a normal blood flow pattern. The patient had no significant comorbidities or any other chronic disorders. There is no family history of joint or back pain.

On examination of the spine, there is exaggerated lumbar lordosis, and mild tenderness at the L4 and L5 spinous process was observed. There is no tenderness on both sacroiliac joints. Her hip joints are normal, but there is mild tenderness on the posterior aspect of both greater trochanters of the femur. Her spinal movements, including forward flexion, extension, lateral flexion, rotation, and neck movements, are painless and have a full range of movement. Her chest expansion is within normal limits. The diagnostic tests for the sacroiliac joint, including the Faber test, pelvic compression, pelvic distraction, pump handle test, and bilateral straight leg raising test, are negative. A clinical diagnosis of chronic lumbosacral strain with bilateral sacroiliac arthralgia was suspected.

**Figure 1:**
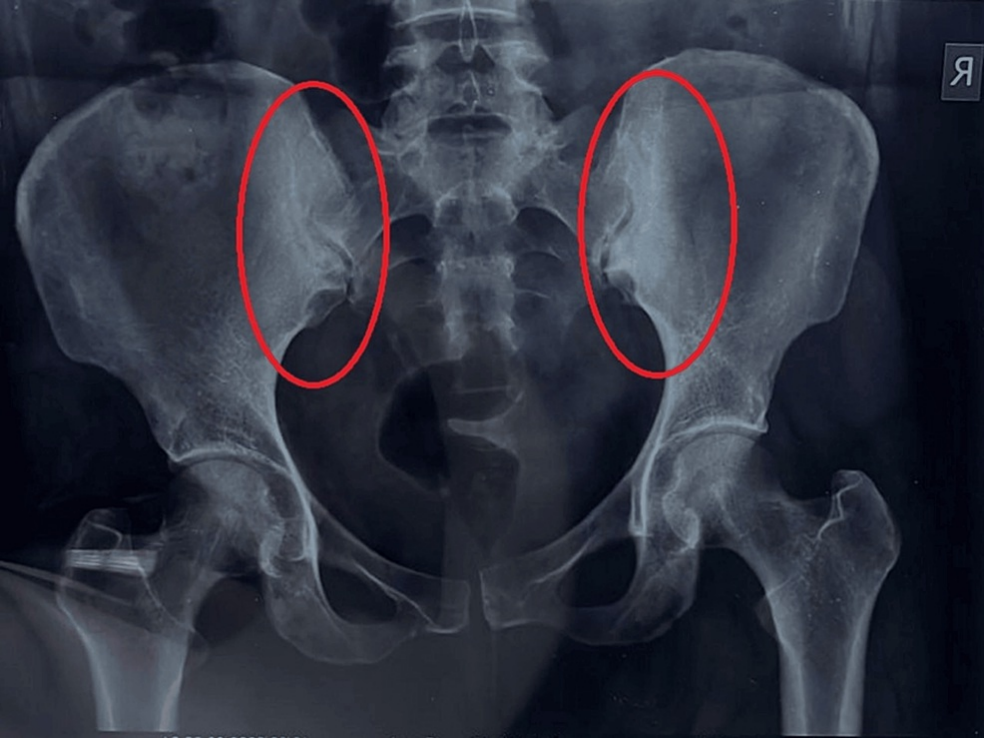
X-ray of the pelvis showing the sclerosis of the iliac part of sacroiliac joints without any erosion, irregularity, or fusion of sacroiliac joints

On X-ray of the pelvis, the sclerosis of the iliac part of the sacroiliac joints without any erosion, irregularity, or fusion of the sacroiliac joints was seen as shown in Figure 1. There was slight irregularity appreciated on the lateral aspect of the greater trochanter, more on the right side of the femur. There was no abnormality seen in the lumbar spine and hip joints.

**Figure 2:**
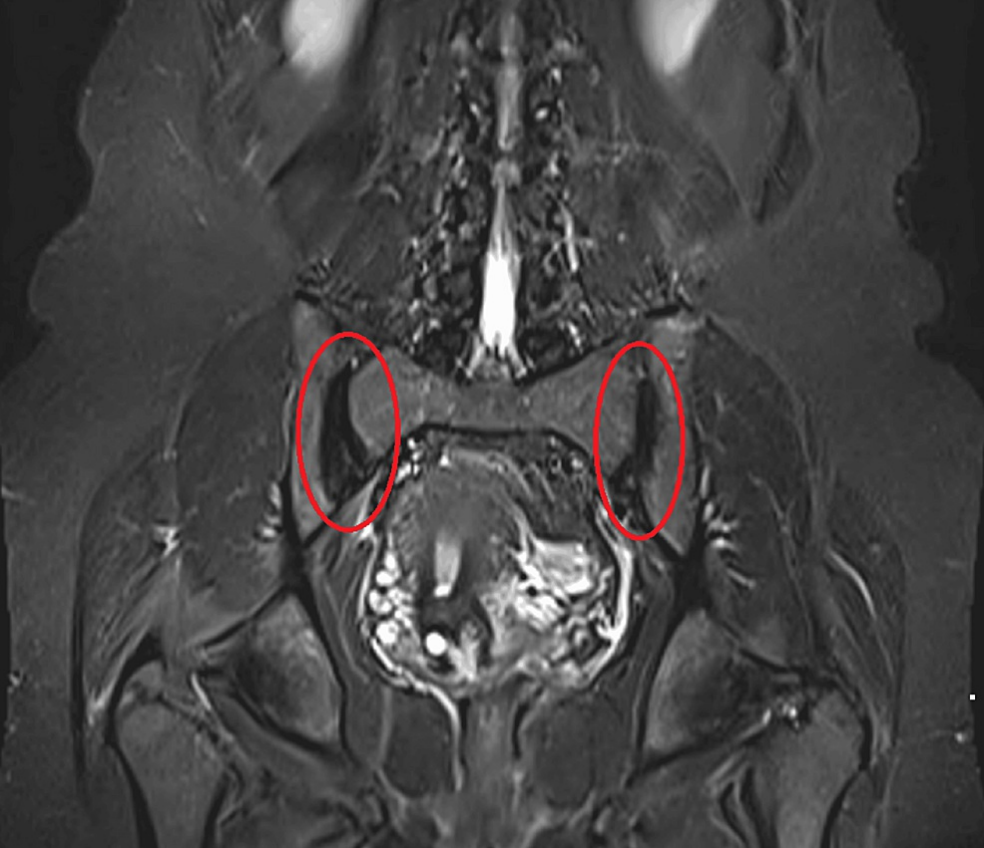
MRI showing focal sclerosis on the iliac aspect of both sacroiliac joints MRI: magnetic resonance imaging

Based on an X-ray, the diagnosis of osteitis condensans ilii was confirmed. On magnetic resonance imaging (MRI) of the lumbosacral spine, focal sclerosis on the iliac aspect of both sacroiliac joints suggestive of osteitis condensans ilii was seen as shown in Figure 2. There was also evidence of a posterocentral annular tear with a diffused disc bulge indenting the anterior thecal sac without nerve root impingement.

Her investigations including blood counts, erythrocyte sedimentation rate (ESR), rheumatoid arthritis (RA), and human leukocyte antigen B27 (HLA-B27) were within normal limits as given in Table 1.

**Table 1:** Blood investigations CBC, complete blood count; Hb, hemoglobin; RBC, red blood cells; PCV, packed corpuscular volume; MCV, mean corpuscular volume; MCH, mean corpuscular hemoglobin; MCHC, mean corpuscular hemoglobin count; RDW-CV, red cell distribution width-coefficient of variation; TLC, total leucocyte count; CRP, C-reactive protein; HLA-B27, human leukocyte antigen B27; RA, rheumatoid arthritis; ESR, erythrocyte sedimentation rate

Name of the test	Result	Reference range
CBC		
Hb%	8.8 g/dL	12.5-16
RBC	4.50 millions/cumm	4.2-5.4
PCV	31.8%	37-47
MCV	71.0 fL	78-100
MCH	19.6 pg	27-31
MCHC	27.7 g/dL	32-36
RDW-CV	18.3%	11.6-14
TLC	8100 cells/cumm	4000-10500
Neutrophils	53%	44-76
Lymphocytes	41%	20-40
Eosinophils	03%	1-6
Monocytes	03%	2-10
Platelet count	322000/cumm	150000-450000
Serum alkaline phosphatase	287 U/L	64-306
RA factor quantitative	2.4 IU/mL	Up to 20
Serum calcium	8.9 mg/dL	8.8-10.5
CRP	12.89 mg/L	Up to 6
Vitamin D3	21.96 ng/mL	Deficiency, <20; insufficiency, 20-<30; sufficiency, 30-100; toxicity, >100
HLA-B27 flow cytometry	Negative	
ESR	16 mm/hour	>30

The patient was explained the nature of the disease and its prognosis. She was taught back extension and abdominal muscle strengthening exercises besides limb, neck, and chest exercises. She was prescribed calcium 500 mg and vitamin D 60000 IU weekly for 10 weeks. At one-year follow-up, she is pain-free and feels no discomfort in the sacroiliac area but is occasionally disturbed due to midline lower back pain.

## Discussion

Osteitis condensans ilii (OCI) is a condition in which there is a disturbance of the normal architecture of the ilium with increased sclerosis of the auricular portion without a corresponding change in the sacroiliac joints or the sacrum. Mechanical stress plays a significant role in developing OCI, even if the reason is unknown [4]. The theory regarding this postulates that the sacrum tends to rotate about a fulcrum situated at the second sacral segment. The strong sacroiliac ligaments resist this rotation. When the tendency of rotation is increased due to acute lumbosacral angle or during pregnancy, causing the softening of pelvic ligaments, additional strain is forced on the ligaments at their attachment to the ilium. The auricular process of the ilium responds to this unnatural strain by bony thickening seen on imaging as sclerosis [5].

Although most patients do not have the HLA-B27, OCI was once thought to be a kind of ankylosing spondylitis. The sclerosis of the auricular region of the iliac bone is the defining feature of this disease. Increased inflammatory markers do not often accompany OCI and are not categorized as inflammatory arthritis, notwithstanding a few unusual occurrences. It is primarily asymptomatic, although, in a few cases, it appears as early-onset low back discomfort that resembles axial SpA [3]. OCI pain is usually bilateral and may radiate to the back of the thighs and buttocks. Point soreness at the sacroiliac joint may or may not accompany the symptoms. Fortin's finger sign is positive when deep pressure is placed posteriorly on the joint, generating discomfort. Hip flexion, abduction, and external rotation result in unpleasant and vague distress. Other provocative tests, including iliac compression, sacral distraction, and thigh/sacral thrust tests, are usually negative [6].

OCI is usually self-limiting; therefore, understanding this fact is crucial when treating the illness. Thus, conservative management such as anti-inflammatory drugs and physical therapy are the cornerstone treatment approaches and are often effective [7]. Reducing the severity and length of pain and stiffness and enhancing the patient's quality of life are the main goals of therapy. Reassuring the patient of the benign nature of the condition and the lack of disease development on clinical and radiographic examinations is the first step in treating OCI [8]. The prognosis is excellent since OCI is not progressing, and conservative treatment is chosen. The management of OCI involves rest, physical therapy, and nonsteroidal anti-inflammatory medicines (NSAIDs). Corticosteroid/anesthetic therapy injections have been used, although the disease is not inflammatory, and in one refractory instance, a surgical core decompression was documented. In rare circumstances, the surgical removal of the affected osteitis bone may also assist in reducing discomfort [2].

Various authors have reported a single case or case series of OCI [3,6]. This case is atypical in the sense that it started one year after the last delivery of the patient. The pain is more on the left side and recurrent, getting relieved on its own. The patient does not have any features of ankylosing spondylitis. The disease was suspected when the patient was investigated for chronic midline lower back pain. Her X-ray of the pelvis showed sclerosis on bilateral iliac surfaces of the sacroiliac joint. MRI diagnosed disc prolapse at L4-L5 and showed the sclerosis of the iliac part of both sacroiliac joints.

## Conclusions

OCI, an uncommon cause of back pain, is often an incidental radiographic finding in asymptomatic individuals, primarily females, after delivery. The condition needs to be differentiated from SpA and inflammatory arthritis. Advanced imaging methods such as MRIs and CT scans are beneficial if there is a solid clinical, radiological, or serological suspicion of another inflammatory illness. Clinicians must be aware of this disease entity and avoid using unnecessary medications in view of its self-limiting nature and good prognosis. Most of the time, its treatment does not require surgery, and it is well controlled with physical therapy, NSAIDs, and rest whenever necessary.
